# Indoor residual spray bio-efficacy and residual activity of a clothianidin-based formulation (SumiShield^®^ 50WG) provides long persistence on various wall surfaces for malaria control in the Democratic Republic of the Congo

**DOI:** 10.1186/s12936-019-2710-5

**Published:** 2019-03-12

**Authors:** Leonard M. Ngwej, Izak Hattingh, Godwill Mlambo, Emmanuel M. Mashat, Jean-Christophe K. Kashala, Françoise K. Malonga, Michael J. Bangs

**Affiliations:** 1 China Molybdenum Co., Ltd./International SOS Malaria Control Programme, Tenke Fungurume Mining, Fungurume, Lualaba Province Democratic Republic of the Congo; 2 School of Public Health, University of Lubumbashi, Lubumbashi, Democratic Republic of the Congo; 3 Faculty of Veterinary Medicine, University of Lubumbashi, Lubumbashi, Democratic Republic of the Congo; 4 Public Health & Malaria Control Department, PT Freeport Indonesia, International SOS, Jl. Kertajasa, Kuala Kencana, Papua 99920 Indonesia; 5 Department of Entomology, Faculty of Agriculture, Kasetsart University, Bangkok, 10900 Thailand

**Keywords:** Clothianidin, Bio-efficacy, Residual activity, Mosquito vector control, Democratic Republic of the Congo

## Abstract

**Background:**

Bio-efficacy and residual activity of SumiShield^®^ 50WG (50%, w/w) with active ingredient clothianidin, a neonicotinoid compound, was assessed using an insecticide-susceptible laboratory strain of *Anopheles arabiensis*. Implications of the findings are examined in the context of potential alternative insecticides for indoor residual spraying in Lualaba Province, Democratic Republic of the Congo.

**Methods:**

Contact surface bioassays were conducted for 48 weeks on four types of walls (unbaked clay, baked clay, cement, painted cement) in simulated semi-field experimental conditions using two different doses of clothianidin active ingredient (200 mg ai/sq m and 300 mg ai/sq m). Additionally, two types of walls (painted cement and baked clay) were examined in occupied houses using the 300-mg dosage. Laboratory-reared *An. arabiensis* were exposed to treated surfaces or untreated (controls) for 30 min. Mortality was recorded at 24-h intervals for 120 h.

**Results:**

Under semi-field experimental conditions, there was no significant difference in mortality over time between the two doses of clothianidin. The mortality rates remained above 60% up to 48 weeks on all four wall surface types. The formulation performed better on cement and unbaked clay with a mean final mortality rate above 90%. Under natural conditions, there was no significant difference in response between baked clay and painted cement walls with a mean final mortality rate above 90%. The insecticide also performed significantly better in natural settings compared to semi-field experimental conditions.

**Conclusion:**

Depending on the type of experimental surface, the residual activity of the two doses of clothianidin was between 28 and 48 weeks based on a 60% mortality endpoint. Clothianidin at 300 mg ai/sq m applied on two house walls (baked clay or painted cement) performed equally well (> 80% mortality) on both surfaces up to week 41 (approximately 9.5 months). Extended bioassay holding periods (up to 120 h) may present with excess natural mortality in the untreated controls, thus complicating analysis.

## Background

The use of vector control in the fight against malaria transmission in sub-Saharan Africa has mainly focused on the mass distribution of insecticide-treated bed nets and periodic indoor residual spraying (IRS) of insecticides [1–4]. When properly performed, IRS is a potent intervention that can greatly reduce indoor adult mosquito vector densities for many months following each repeated application [5, 6]. The reduction in both biting density and survival of vectors lowers indoor malaria transmission risk [3]. When a susceptible mosquito population comes into direct physical contact with an insecticide-sprayed surface, provided exposure time is sufficient, the insect can absorb a lethal dose, thereby decreasing the probability of transmission. IRS has proven effective in lessening the malaria burden in a wide range of operational settings beginning in the malaria eradication era of the 1950s and 60s, and more recently following re-introduction in many meso- to holo-endemic malaria areas of Africa [7]. The World Health Organization (WHO) and others have reaffirmed the importance of IRS as a primary intervention for reducing or interrupting malaria transmission [3, 8].

The increasing emergence and expansion of insecticide resistance in a number of important malaria vector species to one or more of the three commonly available classes of public health insecticides (i.e., pyrethroids, carbamates and organophosphates) threatens the success and usefulness of IRS and treated bed nets in countries involved in transmission control [9–15]. Resistance against various classes of insecticides by *Anopheles gambiae* sensu stricto (s.s.) has also emerged in the Democratic Republic of the Congo (DRC), thereby mandating that resistance management strategies be implemented and supported with regular entomological monitoring [15, 16]. While other vector control strategies are being revisited (e.g., larval source management), and new tools are under development (e.g., transgenic mosquitoes, attractive toxic bait systems, endectocides, etc.) [17–19] for reducing malaria transmission, the use of insecticides remains an essential tool for effective integrated control against malaria [6, 7, 20]. In many malaria-endemic countries, especially those in sub-Saharan Africa, IRS combined with the added protective effects of insecticide-treated mosquito netting, remains an important method of combatting endophilic malaria vectors [21–24].

There has been a compelling call for alternative class insecticides (so-called ‘next generation’ or ‘NG’ products) with different modes of action that can be used in insecticide resistance management and IRS chemical rotation programmes to mitigate selection pressure and possible development of resistance in vector populations [7, 9, 10, 25–27]. Not only is expanding and rapidly emerging resistance a cause for concern, but likewise, the short residual efficacy of most IRS products on the market today hampers effective year-round coverage [28, 29]. Among the alternative newer chemistries evaluated for public health applications, the neonicotinoid, clothianidin, was advanced in development as an IRS candidate against malaria vectors and as a potential insecticide resistance management (IRM) component [3, 25, 30]. More recently, clothianidin-wettable granule formulation was added to the list of WHO recommended insecticides for IRS [31]. Clothianidin is a relatively new compound in the expansive agricultural crop protection market. In 2003, The US Environmental Protection Agency granted clothianidin conditional registration for protection against sucking and chewing insect pests. This class of compounds possesses a novel mode of action, unlike the chemicals typically used in IRS, namely by interrupting the transmission of nerve impulses by irreversibly blocking the nicotinergic acetylcholine receptors on the postsynaptic membrane of the insect nerve call, as opposed to the acetylcholine production on the presynaptic membrane. This is advantageous as a public health insecticide as there would be presumably no cross-resistance mechanisms between neonicotinoids and the other IRS classes currently in use, and they generally show very low mammalian toxicity [32].

Experimental findings reported by the product manufacturer Sumitomo Chemical have shown SumiShield^®^ 50WG, an IRS formulation containing clothianidin, has an effective residual activity under ‘normal’ field conditions ranging from 6 to 9 months [33]. Clothianidin has low excito-repellency activity in mosquitoes, which results in avoidance behaviour, allowing greater toxicant exposure time by the mosquito, increasing the probability of early death. The chemical also has low mammalian toxicity [32–34] and is odourless, thus enhancing its acceptability in populations under vector control coverage. The objective of this study was to evaluate the residual performance of a wettable granule formulation of clothianidin against an insecticide susceptible laboratory strain of *An. arabiensis* when sprayed on different wall materials in both semi-field experimental and natural house conditions in the DRC. The assessment is part of an integrated, evidence-based malaria vector control programme to ensure the most efficient and effective use of IRS chemicals and IRM implementation [25, 30, 35].

## Methods

### Study site

This study was conducted in the town of Fungurume, located in Lualaba Province (formerly Katanga Province), southern DRC. This area experiences perennial high-level transmission of *Plasmodium falciparum* (> 95% of diagnosed patent infections), followed by *Plasmodium malariae* and *Plasmodium ovale*. Transmission intensity shows seasonal and spatial fluctuations in infection risk. The climate is sub-tropical with two distinct seasons (wet and dry), divided near equally into 6-month intervals. The primary malaria vector in the area is *An. gambiae* s.s. with several less abundant species serving as secondary vectors. Since 2008, Tenke-Fungurume Mining (TFM) has routinely implemented a private-sponsored integrated malaria control programme inside a large concession area. Vector control activities have primarily focused on periodic IRS in the vast majority of houses throughout all local communities directly or indirectly impacted by the mining activities.

### Mosquitoes

An insecticide-susceptible laboratory colony of *An. arabiensis* (‘MAL’ strain) was used in all exposure and control assays. The MAL strain is completely susceptible to all currently used classes of public health insecticides in line with WHO recommended concentrations and diagnostic assessments. This strain has been maintained in continuous colony at the Malaria Institute, Tzaneen, Limpopo Province, South Africa since 1994. The colony was introduced to the TFM Vector Control Programme (VCP) insectary in May 2011. All mosquitoes were maintained under controlled insectary rearing procedures (27 ± 3 °C, 60–70% relative humidity), a standardized regime of larval and adult mosquito diets, and handling.

### Insecticide

A single chemical formulation (Lot No. 16940354340Y) was provided as a water-dispersible granule containing 50% (500 mg/kg w/w) clothianidin (SumiShield^®^ 50WG, Sumitomo Chemical Co. Ltd, Japan). Product manufacturing date was December 2014 with a December 2017 expiration. The active ingredient, clothianidin [Chemical formula: C_6_H_8_CIN_5_O_2_S; IUPAC: (E)-1-(2-chloro-1,3-thiazol-5-ylmethyl)-3-methyl-2-nitroguanidine], is a neonicotinoid class insecticidal compound [33].

### Chemical quality assurance

The insecticide formulation was stored in original packaging in dry ambient conditions before use. Sumitomo Chemical Co. provided a certificate of analysis indicating 49.8% clothianidin purity. Based on re-analysis of this formulation, similar samples have shown the product to be stable after more than 3 years storage (pers. commun., Sumitomo). Product was applied in July 2015, approximately 7 months after manufacture (January 2015). The final study observation was in June 2016, approximately 18 months after production. Product was sprayed on wall surfaces as prescribed on the product label. The chemical application equipment was thoroughly cleaned to remove all possible contamination and carefully calibrated to enable as accurate an operational delivery of product as possible on each wall surface. To better ensure as equal a delivery of each dose on surfaces, only one spray unit and the same trained operator was used in the application process.

### Contact surfaces

**Fig. 1:**
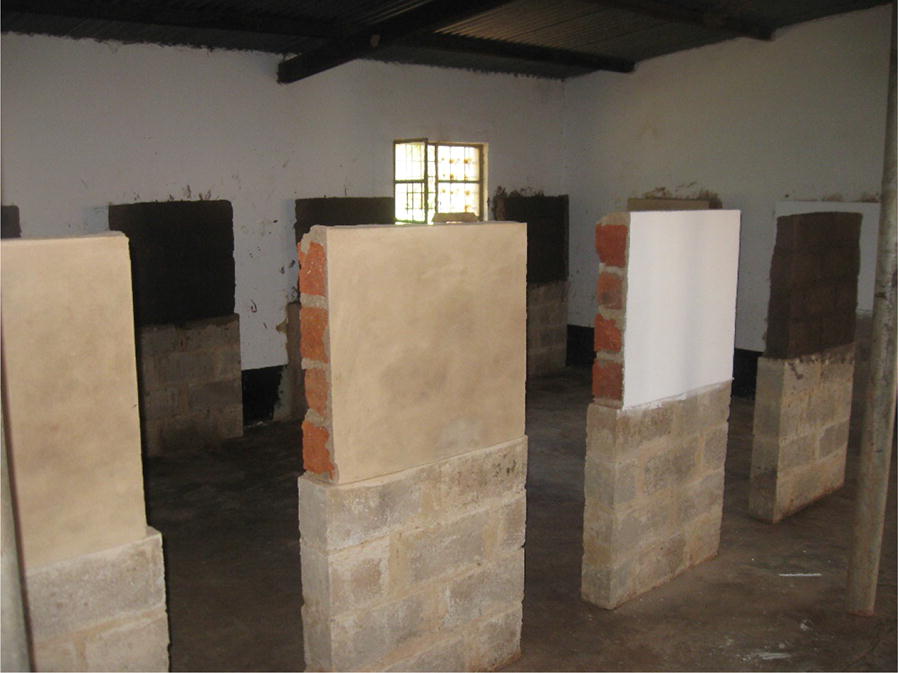
Experimental walls (approximately 0.5 m^2^) showing left to right surfaces of cement, painted cement, and unbaked clay (baked clay surface not shown)

The study was sub-divided into a semi-field, controlled experimental set-up and natural field-based observations in houses. For the experimental portion, four sets of simulated wall surfaces were used, each representative of the most common wall construction materials (unbaked clay, baked clay, cement, painted cement) present in the TFM concession area under IRS coverage. For painted cement surfaces, a white, water-based latex paint was applied to cement block and allowed to air dry. Each wall surface was elevated 80 cm from the cement floor and placed 70 cm apart from one another. Each square surface area was carefully constructed to measure 0.49 sq m (Fig. 1). All experimental walls were located in an enclosed, ventilated, secure space and protected from direct sunlight, moisture and excess dust during the observation period. In the nearby semi-urban community of Fungurume, two local households were selected for voluntary participation. One house had interior painted cement walls, the same paint as applied to the experimental surfaces. The other house had exposed (unpainted) baked clay walls. The two recently constructed houses had not received IRS previously. Voluntary verbal consent was obtained from each house owner with acknowledgement that he/she understood the purpose of the study and that spray surfaces would not be purposefully modified or otherwise adulterated (e.g., cleaned, painted, etc.) by the owners over the course of the observation period.

### Susceptibility assays

Standard WHO insecticide susceptibility tests were performed to confirm the *An. arabiensis* MAL strain mosquitoes were fully susceptible to selected pyrethroid, carbamate and organophosphate class insecticides at the recommended discriminating concentrations [36]. The response to deltamethrin (0.05% concentration), permethrin (0.75%), bendiocarb (0.1%), and pirimiphos-methyl (0.1%) was tested. Insecticide-treated papers were obtained from the Vector Control Research Unit, University Sains Malaysia (Penang, Malaysia). Insecticide-treated filter-paper tube assay kits were used according to WHO procedures using laboratory-reared, 3–4 day-old, sugar-fed, non-blooded, females [36]. Mosquitoes were exposed to insecticides for 60 min with knockdown response observed after 1 h. Mosquitoes were transferred to clean holding cups, provided 10% sugar solution on cotton wool, and held 24 h for recording the final mortality response. A matching control tube without insecticide (carrier compound only) was conducted with each test series. When control mortality was between 5 and 20%, all final contact mortality was adjusted (‘corrected’) using Abbott’s formula [36, 37].

### Insecticide application

For this study, SumiShield^®^ 50WG was applied to various simulated and natural wall surfaces using two different doses of active ingredient, either 200 mg or 300 mg/sq m. Before applying the formulation to test surfaces, contact cone bioassays [38] were conducted on each experimental surface and selected house walls. A minimum of four replicate tests for each wall were conducted to ensure that all surfaces were free of residual insecticides or other chemicals that could influence the assessment of mosquito response to the target chemical.

Following published spray application guidelines [39] all surfaces were sprayed using a standard, calibrated, 15-L capacity Hudson X-Pert^®^ hand-compression sprayer (H D Hudson Manufacturing Co., Chicago, USA) equipped with a hardened stainless steel spray nozzle tip as appropriate to surface characteristics. For spraying, the tank was initially pressurized at an optimal mean pressure of 40 psi (2.76 bars). At 40 psi, the 8002E and 8001E nozzles were pre-tested to ensure output of 760 and 380 mL/min ± 1% variance, respectively.

For preparation of spray solution, 100 g and 150 g of SumiShield was measured out using a calibrated electronic scale and sealed in separate plastic bags by weight. To treat surfaces with either 200 mg or 300 mg ai/sq m, 100 g or 150 g of SumiShield^®^ 50WG was mixed with 10 L of clean water as recommended by the product manufacturer [33]. Experimental surfaces were sprayed with one or the other dose only. House walls were sprayed with the 300 mg dosage only. The spray mixture was applied evenly on each vertical surface and allowed to air dry. The same spray unit and trained operator was utilized for each chemical application to ensure as precise an output per surface area as possible. To prevent cross-contamination, the spray unit was thoroughly cleaned with multiple flushes of clean water between the two different applications, starting with the lower dose. One set of wall surfaces remained blank, sprayed with water only using a new (unused) sprayer, and served as the respective control for each surface type. In houses, a similar type wall was selected in an opposite room to serve as the control. There was no comparator insecticide used in this study.

### Contact bioassays

Nulliparous, non-blood fed, 4–5 day-old *An. arabiensis* females were used in all trials. Standard contact bioassay tests were performed using clean plastic transparent cones based on WHO procedures and analysis [38]. After attaching the cone securely to the wall with masking tape, 10 mosquitoes were placed inside each cone using a mouth aspirator and exposed for 30 min as follows: For experimental conditions, cones were placed at four different locations on the wall (total 40 female mosquitoes) and held undisturbed for 30 min. The exact same locations were used for each test interval to avoid areas having lost some chemical on surface by removal of masking tape. In houses, cones were placed at three different locations on the wall: top section 25 cm below ceiling, middle line of wall, and near the bottom at 25 cm above floor level. Each test interval involved a minimum of three replicates (90 mosquitoes total per wall surface). To reduce potential time-related response bias related to normal mosquito activity rhythms, all contact bioassays occurred during daylight morning hours (08:30–11:30). Air temperature and relative humidity readings were recorded during the 30-min assay.

Immediately following 30-min contact period, all mosquitoes were recorded as either ‘knockdown’ (in a incapacitated or moribund state) or ‘live’ and then carefully removed from each cone and placed in a labelled Styrofoam^®^ holding cup topped with synthetic mesh screen. Each cup was provided a 10% sugar solution soaked on cotton wool placed atop the cup. Holding cups were immediately returned to the laboratory and placed under normal insectary conditions of temperature and relative humidity. Each cup was observed at 24-h intervals recording mortality each period up to 120 h before recording final mortality. Each observation period, dead mosquitoes were removed and sugar solution replaced. During each trial, 30 mosquitoes of identical pre-test condition were used as controls and exposed to untreated surfaces in the experimental design and same sprayed houses.

Final percent mortality was adjusted using matched control mortality at or above 5% at same point in time using Abbott’s formula [37]. Normally, if control mortality exceeds 20%, the paired tests are repeated; however, in some trial instances mortality above 20% was accepted and adjusted accordingly. Repeat trials were not always possible due to limited numbers of suitable conditioned mosquitoes available during certain testing periods. For the same reason, bioassay test intervals also varied in some experimental and natural conditions based on the availability of test mosquitoes. Air temperatures and percent relative humidity were recorded during test exposure periods in experimental and house settings, as well as insectary conditions during the holding period. The WHO threshold (cut-off) guidance for acceptable insecticide performance in cone assays is ≥ 80% mortality [38]. However, due to the greater inherent variability in tests with holding times beyond 24 h, the semi-controlled experimental and field-study endpoint was revised to ≥ 60% mean mortality up to a maximum of 120 h post-contact holding time during two consecutive test periods (i.e., generally a 2-week interval between observations).

### Data analysis

Mortality was the primary outcome measure to determine the residual efficacy of the insecticide. Only control-adjusted numbers were used in the final analysis. Wald Chi square and a generalized linear model (GLM) compared surfaces and concentrations, allowing for response variables that have error distribution models other than a normal distribution. The GLM allowed adjustment of the mosquito mortality based on number of exposed dead mosquitoes compared to the number of mosquitoes at risk of dying, with insecticide concentration, wall type, holding time, and interactions using a log–log link function and a binomial distribution. The estimated effects is expressed by mean mortality with a 95% confidence interval. Wald’s Chi square test tested the effects of insecticide concentration, wall type, time and their interactions. It relied on linear (independent) matched multiple comparisons among estimated marginal means using least significance difference criterion. Kaplan–Meier survival analysis estimated the mean survival duration of mosquitoes during the 5-day holding period and comparisons made using the Mantel–Cox log-rank test. Data analysis utilized SPSS statistical software ver. 23 (SPSS Inc., Chicago, IL, USA). All statistical tests were set at the discriminating level of 5% (*p* < 0.05).

## Results

### Mortality response and residual activity

Clothianidin induced delayed mortality in a pyrethroid-susceptible colony of *An. arabiensis*. Under semi-field experimental conditions, differences between wall type and percent mortality from time of initial application to end of observation was highly significant (*p *< 0.0001), while no differences were seen in final mortality between the two doses (*p *= 0.148). Differences between surface effects (e.g., material, pervious qualities, etc.), time, and wall-time interactions were found highly significant (*p* < 0.0001). Overall, residual life was greater on cement and unbaked clay. With either dose, there was little or no knockdown observed after 30-min contact with treated surfaces.

On painted cement walls, the mean mortality during 48 weeks was 81 and 77.9% for 300 mg and 200 mg, respectively, showing no difference (*p *= 0.439) between doses. Mosquito response to 200 mg showed greater variation than the higher dose up to week 28. A dramatic decline in activity was seen for 200 mg on week 32 (52.8% mortality), while a large reduction in mortality with 300 mg, began on week 35 (57.5%) (Table 1). Interestingly, a substantial rebound for both doses appeared at week 38 (> 90% mortality). This apparent ‘recovery’ was short lived however, with both showing precipitous declines in mortality to week 46 (< 10% kill). However, on week 48 (final test period), there was a large upsurge in activity seen with 200 mg and a slight increase at 300 mg. Increased activity was seen for both doses on all test surfaces on final week 48.

**Table 1:** Corrected mean percent mortality rates of *Anopheles arabiensis* following 30 min contact with two doses of clothianidin wettable granule formulation applied on different wall surfaces under experimental conditions

Surface	Dose (mg/m^2^)	Weekly percent mortality^a^																	
		0	2	4	6	8	10	14	18	20	22	28	32	35	38	41	43	46	48
Painted cement	300	100	100	100	100	100	88	100	92.5	90	92.5	78.6	100	57.5	94	47.5	18.4	5	9.4
Painted cement	200	100	87.5	67	94	100	70	100	72.5	85	100	100	52.8	42.5	91	55	44.7	8.3	68.8
Baked clay	300	100	92.5	83.7	80.6	100	34.4	100	45	52.5	100	42.1	78.6	51	100	0	0	2.5	26.5
Baked clay	200	100	100	100	97.2	100	90.6	100	95	85	100	83	100	88.5	100	68.4	0	5.3	52.9
Unbaked clay	300	100	100	100	100	100	NT	100	97.5	100	100	100	100	100	100	100	50	60	96.9
Unbaked clay	200	100	100	100	96.9	100	NT	100	100	97.5	100	100	100	100	100	96.1	73.3	47.5	87.5
Cement	300	100	100	100	100	100	100	100	100	100	100	100	100	100	100	100	68.4	66.7	81.1
Cement	200	100	100	100	100	100	100	100	100	100	100	100	100	100	100	100	84.2	86.1	94.7
Mean temp (°C)		20.2	20.3	19.2	21.3	23.5	23.5	26.5	24.8	24	24.6	23.9	23.1	23.7	24.5	24.3	24.1	25.1	21.4
Mean % humidity		50.3	49.5	43.5	42.8	48.3	41.8	42	70.3	74.5	71.8	82	84.3	82.3	74.3	70.5	65.5	49.5	58.5

*NT* not tested

^a^Final mortality adjusted if control mortality > 5%

On baked clay surfaces, the mean mortality over the 48-week period was 85.1% for 200 mg and 64.6% for 300 mg; however, this difference was not significant (*p *= 0.065). The percent mortality for the lower dose remained above 80% up to week 38, and above 60% up to week 41. The higher dose showed inordinately high fluctuations between observation periods; for example, varying from 34.4% in week 10 to 100% mortality in weeks 22 and 38 (Table 1). No mortality was recorded in weeks 41 and 43 for 300 mg, whereas 200 mg dropped to zero in week 43. A very small recovery in toxicity was seen for both doses in week 46. As observed on other surfaces, there was a marked increase in mortality for both doses on week 48, and greater recovery seen with 200 mg.

For unbaked clay surfaces, mortality was consistently above 95% through week 41 for both dosages (Table 1). The mean percent mortality for 200 and 300 mg was 95.6 and 95.9%, respectively, over the entire 48 weeks. Although 200 mg showed a marked decline (below 60%) at week 43, followed by a near identical reduction for 300 mg at week 46, there was no significant difference in overall response between the two (*p *= 0.471). As with other surfaces, week 48 showed a notable rebound in response for both concentrations, with a final mortality of 87.5 and 96.9% for the lower and higher doses, respectively.

On cement surfaces, mortality responses were also uniform throughout the study period (Table 1). Both doses produced 100% mortality up to week 41. There was no significant difference (*p *= 0.826) in mean mortality (200 mg = 98.5% and 300 mg = 96.5%) through week 48 and neither dosage resulted in mortality dropping below 60% during the entire study period. On weeks 43 and 46, there was a simultaneous drop in response for both doses with a subsequent rebound in activity at week 48 (200 mg = 94.7% and 300 mg = 81.1%).

**Fig. 2:**
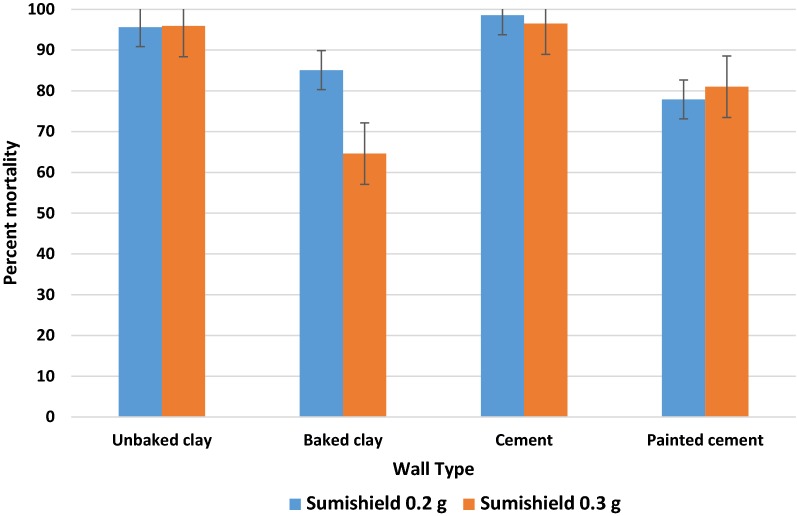
Comparison of mean percent mortality of mosquitoes exposed to 2 concentrations of clothianidin wettable granule on 4 different wall surfaces under experimental conditions from 2 to 48 weeks

**Fig. 3:**
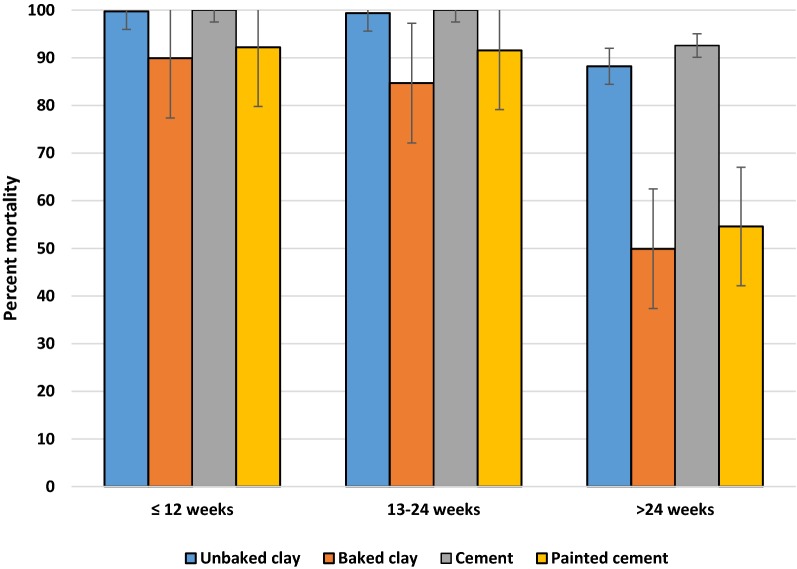
Comparison of effective residual life (percent mortality) of clothianidin wettable granule on 4 experimental wall surfaces over three-time intervals (up to 48 weeks)

When comparing surfaces, unbaked clay and cement surfaces provided the greatest activity over time, followed by painted cement and baked clay (Fig. 2). In general, all walls performed above the 60% mortality study threshold during the 48-week observation. A comparison of the residual life of the product on different surfaces divided between three different time intervals (< 12, 13–24, and > 24 weeks) showed a significant change in scored mortality (*p *= 0.0028) following week 24 on all surface types (Fig. 3). The reduction in mean mortality was more pronounced for baked clay and painted cement surfaces.

**Fig. 4:**
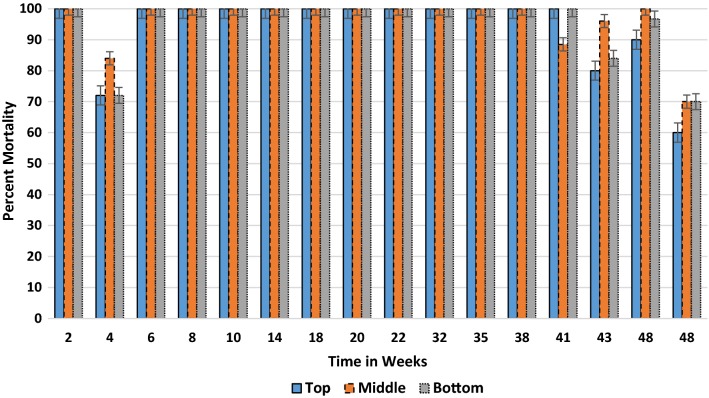
Residual efficacy (percent mortality) of clothianidin (0.3 g/m^2^) wettable granule under natural conditions at three wall locations on painted cement surface

**Fig. 5:**
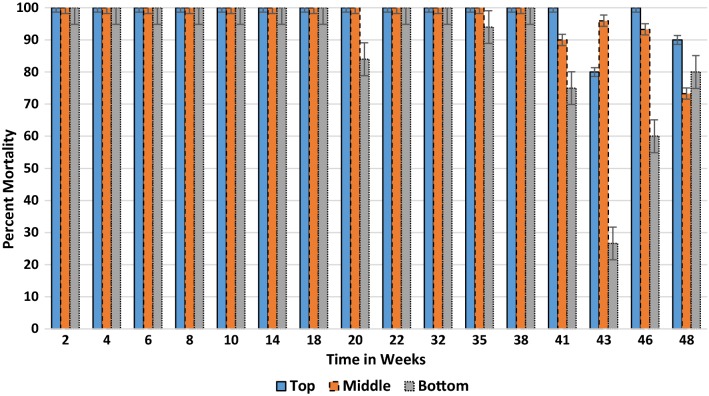
Residual efficacy (percent mortality) of clothianidin (0.3 g/m^2^) wettable granule under natural conditions at three wall locations on baked clay surface

Under natural conditions, the residual efficacy of the 300 mg/sq m WG formulation inside houses on painted cement and baked clay surfaces were tested 1 day after the experimental surfaces. During the 48 weeks, the mean mortality for both wall types remained above 60% and comparable between both surfaces (*p *= 0.260) with a mean of 96.1% for baked clay and 94.8% for the painted cement wall (Table 2). Strong responses were generally produced each test period up to week 43. The residual efficacy was assessed based on three different locations (top, middle and bottom) on the sprayed wall for painted cement (Fig. 4) and baked clay (Fig. 5). There was no significant difference in mortality response by location on wall for painted cement (*p *= 0.99) or baked clay (*p *= 0.08); however, the latter surface produced far more variability in final mortality from week 41 to week 48.

**Table 2:** Corrected mean percent mortality rates of *Anopheles arabiensis* following 30 min contact with 300 mg/m^2^ dose of clothianidin wettable granule formulation on baked clay and painted cement walls under natural field conditions

Surface	Weekly percent mortality^a^																
	0	2	4	6	8	10	14	18	20	22	32	35	38	41	43	46	48
Painted cement	100	100	76	100	100	100	100	100	100	100	100	100	100	96.2	86.7	95.6	66.7
Baked clay	100	100	100	100	100	100	100	100	94.7	100	100	98	100	88.3	75.5	84.4	81.1
Mean temp (°C)	20.3	21	20	22.8	24	25.9	25.4	24.5	24.8	23.5	25	24.5	25.3	24.7	23.2	22.4	21.8
Mean % humidity	53.5	56	45.5	43.3	43.3	62.3	44.8	74	79.8	79	80.8	80.5	81	72.8	63.8	56.8	55.5

^a^Final mortality adjusted if control mortality > 5%

**Fig. 6:**
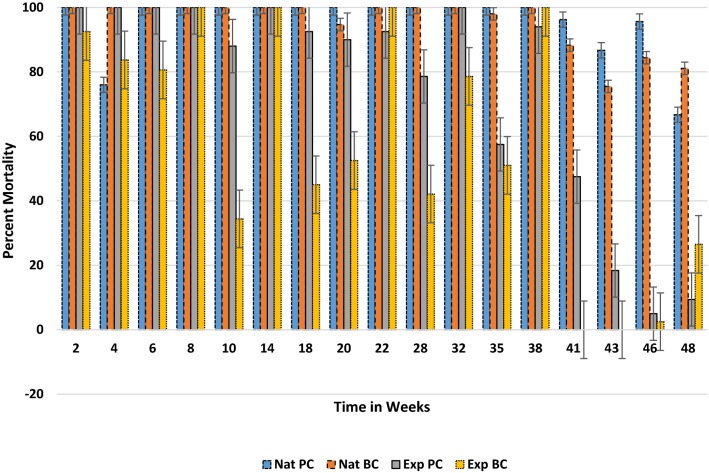
Comparison between natural and experimental wall conditions for the residual efficacy (percent mortality) of clothianidin (0.3 g/m^2^) wettable granule on painted cement and baked clay surfaces

The comparison of residual efficacy between natural and experimental conditions on painted cement and baked clay surfaces (Fig. 6) found mortality responses significantly stronger and more consistent in the house compared to experimental conditions (*p *= 0.034 and 0.002, respectively). For both experimental surfaces, greater variability in mortality between test periods occurred throughout the study, particularly in weeks 41 through 46 where mortality declined dramatically, in particular baked clay (no mortality after 120 h in weeks 41 and 43). This was followed by a gradual but low recovery by week 48 (< 10% painted cement and < 30% baked clay). Conversely, both surfaces in houses performed above 65% at end of the study.

**Fig. 7:**
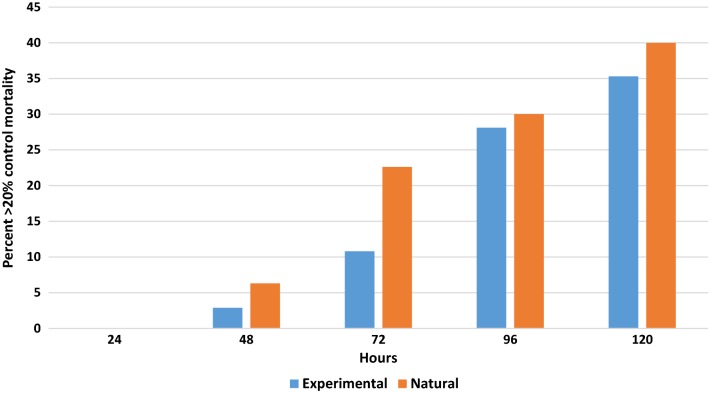
Comparison of mean cumulative percent control mortality each 24-h interval exceeding 20% mortality from day 1 to week 48 under natural and experimental conditions

Lastly, the cumulative control mortality during the 120 h holding period was compared between house and experimental conditions (Fig. 7). The prolonged holding periods found final mortality above 20% in 47 of 313 assays (15%) in the experimental format and 21 of 132 assays (15.9%) from houses. There was an increase in control mortality above 20% with each succeeding 24-h period, beginning with no mortality surpassing 20% during the first 24 h for both experimental and natural settings and rising to 35.3 and 40% mortality, respectively by 120 h.

### Time to mortality estimates

**Fig. 8:**
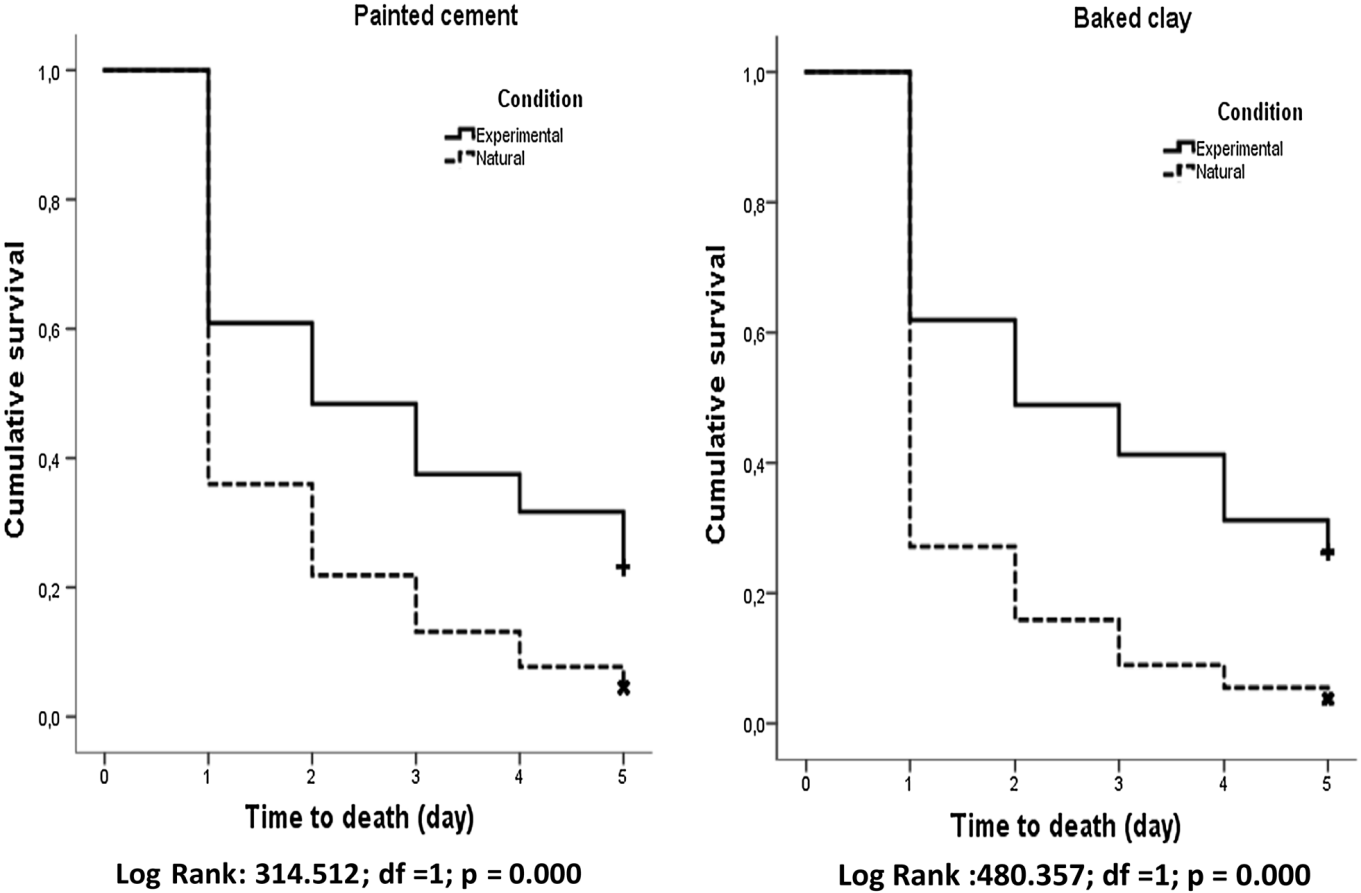
Survival curves of painted cement and baked clay walls between experimental and natural conditions

**Fig. 9:**
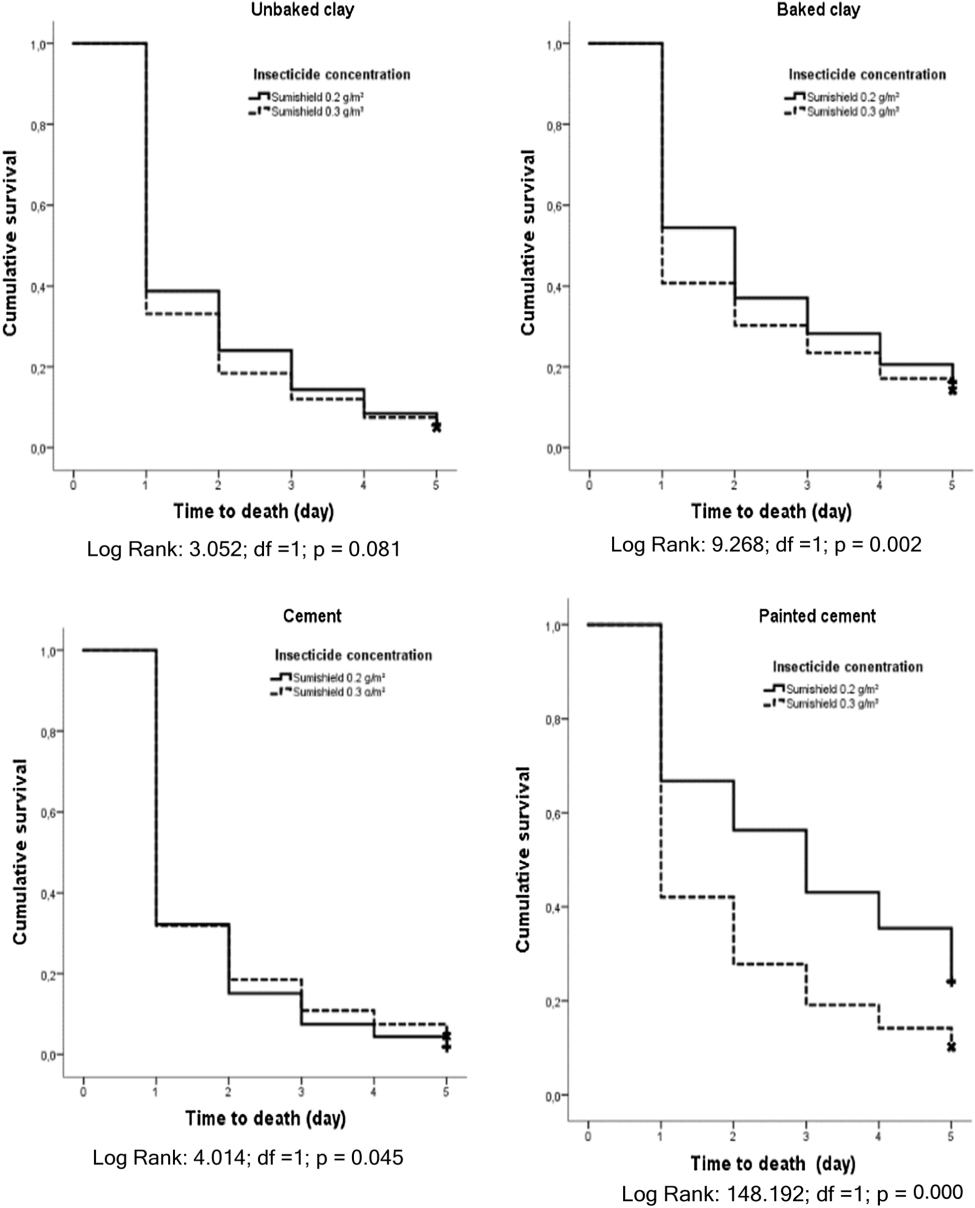
Survival curves of unbaked clay, baked clay, cement and painted cement walls under experimental conditions

Mortality for each bioassay was recorded every 24-h interval up to 120 h (5-day holding period). Final percent mortality was determined at 120 h, unless all specimens died beforehand. Final mortality was adjusted to matching control mortality if ≥ 5%). Survival analysis was used to estimate mosquito survival up to 120 h (Figs. 8, 9). For experimental unbaked clay surfaces, the mean time to death was 1.711 days (~ 41 h) for 300 mg and 1.856 days (44.5 h) at 200 mg, showing no significant difference (*p *= 0.081) between doses. For baked clay, all mosquitoes succumbed to 300 mg at 3.262 days (78.3 h) and 2.403 days (57.7 h) for 200 mg, a significance difference (*p* < 0.001) between doses. Mosquitoes exposed to treated cement surfaces died at 1.688 days (40.5 h) at 300 mg and slightly less for 200 mg at 1.593 days (38.2 h), showing no significant difference (*p *= 0.05). For painted cement, 300 mg resulted in death by 2.553 days (61.3 h) and 3.016 days (72.4 h) for 200 mg, differences that were very significant (*p *= 0.003).

Under house conditions, when examining spray locations, mosquitoes exposed at the bottom section of baked clay died at 48 h (2 days), those at the mid-section were killed by 35 h (1.46 days), while those at the top of the wall were dead at approximately 30 h (1.26 days), differences that are significant (*p* < 0.001) between wall positions. The reason for response differences on various sections of the wall is unclear but may be due to differences in human activity (contact proximity) with surfaces or possibly differences in deposition of insecticide between wall sections. For the painted cement surface, the mosquitoes exposed at the bottom of the wall died at 44.3 h (1.846 days), mid-section before 42 h (1.746 days) and those at the top before 42.5 h (1.769 days). Unlike baked clay, there was no significant difference in mortality between the three wall locations (*p *= 0.506).

There was a significant difference (*p* < 0.001) on baked clay between experimental surfaces and house where mean mortality was much quicker in the latter (37.8 vs 68 h). Likewise, a similar separation in mean mortality (*p *< 0.001) was seen for painted cement in the house (~ 43 h) vs experimental surface (~ 67 h).

## Discussion

In the DRC, a clothianidin-based formulation showed marked persistence and bio-efficacious residual activity when applied on different wall surfaces with varying characteristics. This study examined the residual activity of two doses of clothianidin (200 mg/sq m and 300 mg/sq m) formulated as a wettable granule (SumiShield^®^ 50WG) when applied to various inert (i.e., non-toxic) surfaces using conventional hand-compression application equipment and techniques. The study was divided into two formats. The semi-field experimental portion used four specially constructed surfaces (approximately 0.5 sq m surface size), each simulating common wall surfaces used to construct homes in southern DRC. Surfaces of unbaked clay, baked clay, cement, and painted cement were sprayed with one or the other dose (treated) or with clean water serving as blank control using standard hand application procedures. The second format used the higher dose applied inside two houses with either painted cement or baked clay walls. An untreated wall surface in each house served as the blank control. Using contact cone bioassays and an insecticide-susceptible *An. arabiensis* colony, comparisons were made between different surfaces and two doses in the experimental format. In houses, comparisons were made between the two different wall surfaces. Finally, a comparison was made between matching experimental and natural wall surfaces at the 300-mg dose. The effective residual activity, measured as post-exposure mortality over five consecutive 24-h periods (total 120 h) was recorded at intervals of between 2 and 6 weeks for up to 48 weeks (337 days) before study conclusion.

The study findings revealed clothianidin provided an exceptionally prolonged effective persistence on all tested wall surfaces compared to the average residual activity of many other commercial insecticidal products used for IRS [31, 40]. In semi-field and house conditions, the insecticide effectively killed insecticide-susceptible female *Anopheles* based on a pre-determined cut-off at or above 60% mortality throughout the study period. This lower cut-off contrasts from the conventional guidance for determining the time (weeks or months) of ‘acceptable’ residual efficacy at ≥ 80% [38]. The basis or rationale for this particular WHO cut-off value is unclear and appears arbitrarily derived for the most part. The importance and merit of a particular residual effectiveness would be relative and dependent to the operational and epidemiological circumstances of a particular area.

Using a study endpoint of 60%, under experimental conditions, the mean residual activity for 300 mg was 38 weeks on painted cement and up to 48 weeks for unbaked clay and cement only surfaces. The residual activity on baked clay was more difficult to ascertain due to the large fluctuations in mortality recorded between sequential test intervals. There is no clear explanation for the sometimes substantial and contradictory variance in responses over time, particularly at the higher dose on baked clay and lower dose on painted cement. Even more perplexing was the reduction (in some instances dramatic) in mortality towards the end of the study with a subsequent ‘recovery’ in toxicity effects during the last 2 weeks of study observation. It would appear subtle environmental factor(s) might have played a role influencing mosquito response, either at the time of test exposure or during the extended holding periods. A less likely explanation is possible variations in colony fitness between test generations. As example, control mortality during these fluctuations did not differ significantly between one test interval and the next.

The study presents some limitations in design and interpretation of findings. For instance, in some cases the limited insectary capacity did not allow a sufficient number of mosquitoes of the same age range to be available for every assay. Therefore, sometimes the alignment on the planned scheduled timing for each test (every 2 weeks) was not possible. Furthermore, periodic reduced insectary output precluded cone tests on wall surfaces in houses. Under natural conditions, it was not possible to find previously unsprayed (IRS) houses with the representative four wall types used in the experimental design. Only unsprayed baked clay and painted cement houses were tested and where household owners allowed the trials to be conducted. Given the long post-exposure holding period up to 120 h, in some instance test findings were retained where the control mortality exceeded 20% by applying the Abbott’s formula.

The observed differences in response were associated with the surfaces tested and not believed directly attributed to test conditions per se (i.e., time of test, mosquito age, ambient temperature, relative humidity) as these were controlled as best as possible in the experimental format and to a more limited extent in the houses. However, the effect of even subtle differences in physical parameters could have had played a substantial role in measured outcomes. Under experimental conditions, maintenance of uniform room temperature and humidity between tests was not always possible as time of tests where not always uniform. The impact of varying environmental temperature during and following contact may have influenced toxicity responses due to a temperature coefficient effect of the active ingredient [41, 42] or having effected the overall fitness (longevity) of the test mosquitoes, thus potentially compromising comparisons between test periods. For instance, imidacloprid, a related neonicotinoid compound, has shown a positive temperature coefficient with increased toxicity in insects as ambient temperature rises [43]. Whether clothianidin also shows a positive temperature coefficient is unclear and not examined in this study.

In this study, mosquito mortality was recorded up to 120 h following insecticide exposure. The mean relative humidity in the insectary during the holding period was around 65%, lower than what typically is considered optimum (75% ± 5), thus a lower humidity may have contributed to the cumulative higher mortality in the control test samples. Because of the prolonged holding time (fivefold longer than the standard 24-h susceptibility test), it would be advised to use younger mosquitoes (e.g., 2–3 day-old females) to possibly increase probability of greater survival and circumvent excess control mortality during holding.

Lastly, as with other study designs [40, 44–46], no attempt was made to quantify the actual concentration of active ingredient deposited on the sprayed surfaces, for example, by using High Performance Liquid Chromatography methods [47–49]. Given reports of high variability between target and actual dose applied to surfaces, often ± 25% above or below acceptable target range [50], this design limitation potentially affected test findings and interpretations. However, every effort was made to apply as high quality and uniform a spray concentration as possible on each surface according to best practice and product instructions, thus reflecting the spray applications as they would naturally occur under normal conditions.

Under experimental conditions, there was no significant difference in mosquito response between the two concentrations on the four types of wall surfaces. This suggests that 200 mg/sq m is likely as effective to control and comparable to the 300-mg dose. For 200 mg, 60% mean mortality was scored at 28 weeks on the painted cement surface, 41 weeks on baked clay, and at least 48 weeks on unbaked clay and cement only surfaces. The 0.3-g/sq m concentration was effective up to 38 weeks on the painted cement surface and at least 48 weeks for the unbaked clay and cement surfaces. In houses, the residual activity of 300 mg was at least 48 weeks for both painted cement and baked clay surfaces. Using the conventional WHO threshold of 80% mortality, the residual activity of 0.3-g/sq m concentration lasted up to 38 weeks on the painted cement surface, and respectively 41 weeks on the unbaked clay and the cement surface. For the 0.2 g/sq m, the insecticide lasted up to 28 weeks for the painted cement surface, 38 weeks for the baked clay surface, and respectively 41 and above 48 weeks for the unbaked clay and the cement surfaces. As for the 60% cut-off, the residual activity of the 300 mg was at least 48 weeks in the two houses. Variations in persistence of chemicals on various surfaces is reported elsewhere [44, 49, 51]. The residual life of clothianidin exceeded 7 months, both on laboratory-susceptible and pyrethroid-resistant *An. gambiae* in semi-field (experimental huts) conditions in Benin [33]. In another evaluation in Benin, using a combination formulation of 200 mg clothianidin and 25 mg ai/sq m deltamethrin, mosquito mortality remained above 80% for 8–9 months on mud and cement walls, concluding the mixture potentially provides prolonged control of pyrethroid-resistant populations of *An. gambiae* [45]. A recent study examining IRS with SumiShield WG in India found 300 mg ai/sq m effective for up to 6 months against pyrethroid-resistant *An. culicifacies*, and both operationally feasible and safe [52]. Agossa et al. [40] examined the efficacy of SumiShield 50WG at 300 mg ai/sq m using experimental huts under semi-field conditions in Benin against a susceptible strain of *An. gambiae* and pyrethroid-resistant populations of wild-caught *An. gambiae* sensu lato. On smooth cement surfaces, the formulation gave an overall mean mortality of 91.7% at 120-h holding time with the susceptible strain showing greater than 80% mortality for 32 weeks after spraying. Both knockdown effect and induced exophily for clothianidin was very low compared to deltamethrin.

The prolonged residual efficacy of clothianidin is attributed to its natural degradation over time, metabolites that are also toxic to insects [53]. In agriculture, where this chemical is more commonly used [53], the half-life in soil has been measured at between 495 and 990 days [54]. Having a relatively long residual life is one important factor in selecting an insecticide for IRS, depending on the site-specific epidemiological circumstances and situations where only one carefully timed spray round per year is possible due to cost or other considerations [55].

In this study, under experimental conditions, the unbaked clay surfaces showed a residual life > 90% up to week 41. This is particularly encouraging as this type of wall surface is considered more pervious (porous) compared to cement, cement that has been plastered and/or painted, baked clay, and wood substrates, thus reducing the residual effective life of the insecticide because of sorption of active ingredient preventing contact with mosquito tarsi [56, 57]. For both doses, the residual life on the unbaked clay surface was above expectations and a very promising finding. Unbaked clay remains a common building material in many rural African areas [57] and accounts for over 40% of structures inside the TFM concession [TFM Malaria Control Programme 2016, unpublished report].

Natural walls and conditions provided better results for persistence and mosquito morality than with experimental surfaces for both cement painted and baked clay materials. This is also encouraging as one of the factors that can affect an insecticide’s efficacy and bioavailability over time is human activity inside a sprayed house. However, there is confidence the results obtained from the experimental surfaces are representative of those walls present in the community. The experimental surfaces were constructed as close as possible to those used to construct houses, while the application equipment and spray procedures were identical to IRS provided in the community.

Throughout the study period, mosquitoes died on average between 1.5 and 2 days following exposure on treated unbaked clay and cement walls, and between 2 and 3.5 days for baked clay and painted cement walls under experimental conditions. In houses, mosquitoes died between 1 and 2 days after exposure to treated baked clay walls and between 1.5 and 2 days for the painted cement surfaces. Examining effects of spray location in houses found the bottom of the sprayed wall had more delayed mortality than the middle and top sections. In part, this is understandable as the bottom of the wall is typically more exposed to human and other animal activities (i.e., contact and removal of active ingredient from sprayed surface) than the middle and top areas. When comparing experimental and house conditions, mosquitoes died between 1.5 and 2 days in houses with baked clay and painted cement walls, while mortality was more delayed up to a day longer on the experimental walls (between 2.5 and 3 days).

The study findings revealed that the majority of mosquitoes died between 1 and 3.5 days post-exposure. The speed at which an insecticide acts on a susceptible mosquito, unless excessively slow, should not be a limiting factor in control of malaria transmission as the parasite takes generally up to 10 days or longer to develop in the mosquito from time of imbibing gametocytes before being transmissible to another person. A slower-acting insecticide may be preferable as it reduces the selection pressure on evolving resistance [58]. Chlorfenapyr (a pyrrole class insecticide) is another slower-acting chemical being advocated for use in IRM strategies where mortality can also be delayed up to 72 h post exposure [25, 36, 59, 60].

With the increasing emergence of resistance in mosquitoes to many of the current insecticides used for IRS, there is a critical need for alternative insecticides with different modes of action for replacement or rotation with traditional insecticides [9]. As a broad-spectrum neonicotinoid, it acts selectively as central nervous system agonist of the postsynaptic nicotinic acetylcholine receptors molecular target site [61]. As a result, there is no cross-resistance to conventional public health insecticide classes, which include chlorinated hydrocarbons, organophosphates, carbamates, or pyrethroids [62]. Additionally, clothianidin has much lower toxicity in vertebrates as there is a smaller number of nicotinic receptors with high affinity to neonicotinoids compared to susceptible insects and other invertebrates [53].

IRS alone or in combination with insecticide-treated mosquito nets is recognized as a primary and effective vector control intervention for the reduction of malaria transmission in many areas in Africa [60, 63–66]. Therefore, clothianidin represents a significant advancement for future insecticide-based interventions to combat resistance. It is also important that clothianidin-based products be used prudently to mitigate the development of resistance, a development that has been reported in some agriculture insect species [61, 62]. Residual activity might be extended even further using newer formulations such as encapsulation of the active ingredient in suspension. Clothianidin can be used alone or in combination with other chemicals [45, 52, 61, 67, 68]. Clothianidin, alone or in combination with another insecticide, has successfully undergone Phase II and III studies under the former WHO Pesticide Evaluation Scheme for use in IRS [52, 67, 68]. More recently, SumiShield^®^ 50WG was advanced by the WHO prequalification programme for vector control products (PQ approved 25/10/2017, PQT-VC Reference 001-001) at a target dosage of 300 mg ai/sq m (http://www.who.int/pq-vector-control/prequalified-lists/sumishield50wg/en/, http://www.theglobalfund.org/media/5857/psm_indoorresidualsprayirsgf_list_en.pdf?u=636679306830000000).

Currently, there is no established or recommended operational discriminating concentration for clothianidin susceptibility testing to measure mosquito response. Moreover, the testing format to assess mosquito response remains standard WHO test procedures that requires a post-exposure holding time (minimum 24 h). Because clothianidin induces delayed mortality that requires a holding time of up to 120 h after exposure, assays must consider the implications of increased natural mortality over time (i.e., daily survival rate) seen in the comparison controls. Adjusting final mortality responses using Abbott’s formula has been long prescribed [37], so long as control mortality remains between 5 and 20%, otherwise test findings are typically discarded. For control mortality that exceeds 20%, correction adjustments and test validity regards actual treatment effect become more problematic. As control mortality increases naturally over time, this can greatly influence the evaluation of a slower-acting ingredient based on a control-adjusted mortality. In this study, 15% of experimental and 15.9% of control trials exceeded 20% mortality at or before 120 h. Nevertheless, assays exceeding 20% control mortality were not discarded in the study analysis in as much as natural survival rates remain valid epidemiologically and that any excess mortality attributed to clothianidin would contribute to a further reduction in transmission risk.

The continuous and impudent use of insecticides can exert strong selection pressure on a population to develop, maintain and intensify phenotypic resistance to chemicals [69]. Yet the actual consequence of insecticide resistance on mosquito-borne pathogen transmission remains poorly understood as other factors encompassing epidemiological, vector physiology and age, and environmental conditions are often lacking in order to measure the true impact of an insecticide on a natural population with or without phenotypic expression of resistance [70, 71]. Therefore, depending on the circumstances, insecticide resistance by itself may not necessarily lead to vector control failure. Likewise, an insecticide, like clothianidin, can still function to reduce risk of transmission by delayed mortality, provided it decreases average vector survival below the incubation period of the pathogen [72, 73].

Vector control operations remain focused on using the most effective and efficacious means possible to control malaria mosquitoes. Ideally, that would include chemicals that show strong toxic or excito-repellent responses against target species. Well-designed studies are required to assess both entomological and epidemiological impact of alternative control methods against vector populations to reduce longevity or modify behaviour [74] that impact vector capacity and disease incidence. Such evidence in the context of local vector ecology and epidemiologic interactions [75] is required to derive better site-specific solutions. Although semi-field experimental designs are useful for initial assessment purposes, as seen in this study, extrapolating those findings to natural conditions requires circumspection. The evaluation of insecticides in actual houses are likely more informative but also present with greater complications and variability compared to more controlled settings. Such evidence in the context of local vector ecology and epidemiologic interactions [75] is required to derive site-specific solutions to help guide control programmes.

## Conclusions

Among alternative chemistries that have been evaluated recently for public health applications, the neonicotinoid, clothianidin, has been advanced in development, from candidate to a WHO PQ-approved product formulation, for use in IRS against malaria vectors and as a component for insecticide resistance management to extend the life of insecticidal control. This study evaluated the residual performance of a wettable granule formulation of clothianidin against an insecticide susceptible laboratory strain of *An. arabiensis* when sprayed on different wall materials in both experimental and field conditions in the DRC. At 200 mg ai/sq m clothianidin, SumiShield WG formulation provided greater than 90% mortality on different sprayed surfaces up to 38 weeks (~ 9 months) using semi-field experimental conditions. Clothianidin at 300 mg ai/sq m applied on two house walls (baked clay or painted cement) performed equally well (> 80% mortality) on both surfaces up to week 41 (~ 9.5 months). Further evaluation using this compound and formulation in different locations and settings is encouraged, to include effectiveness (alone or in combination with other active ingredients) against native, insecticide-resistant anopheline populations. Moreover, its inclusion as an alternative chemical for use in risk mitigation and IRM strategies requires urgent attention [30, 35].

## Abbreviations

ai: active ingredient
DRC: Democratic Republic of the Congo
GLM: Generalized Linear Model
IRM: Insecticide Resistance Management
IRS: indoor residual spraying
IUPAC: International Union of Pure and Applied Chemistry
NG: next generation
NT: not tested
SPSS: Statistical Package for the Social Sciences
TFM: Tenke Fungurume Mining
VCP: Vector Control Programme
WG: water-dispersible granules
WHO: World Health Organization
